# The Visual Effectiveness and Cost‐Effectiveness of Vitrectomy and Membrane Peeling for Primary Idiopathic Epiretinal Membranes (iERMs): A Systematic Review

**DOI:** 10.1155/joph/5546933

**Published:** 2026-01-04

**Authors:** Kodchawan Doungsong, Hoi To Kwong, Teresa Sandinha, David H. Steel, Ned Hartfiel, Rhiannon Tudor Edwards

**Affiliations:** ^1^ Centre for Health Economics and Medicine Evaluation, Bangor University, Bangor, UK, bangor.ac.uk; ^2^ University of Liverpool Department of Eye and Vision Science, University of Liverpool, Liverpool, UK, liv.ac.uk; ^3^ Royal Liverpool University Hospital Saint Paul’s Eye Unit, University of Liverpool, Liverpool, UK; ^4^ Newcastle University Biosciences Institute, Newcastle University, Newcastle Upon Tyne, UK

**Keywords:** cost-effectiveness, health economics, idiopathic epiretinal membrane, systematic review, vitrectomy

## Abstract

**Introduction:**

Primary idiopathic epiretinal membrane (iERM) is a common finding in people aged 50 years or over. It is treated with vitrectomy. There are no definite criteria for when to conduct surgery, and outcomes are variable. This systematic review assessed the effectiveness and cost‐effectiveness of vitrectomy surgery for iERM.

**Material and Methods:**

Medline, Embase, Cochrane Library and Scopus were searched. Prospective cohort studies, randomised controlled trials (RCTs) and health economics studies published from 2000 to May 2024 were included. The efficacy endpoint was the mean change in best‐corrected visual acuity (BCVA) from baseline. Summarising effect estimates were used to synthesise results.

**Results:**

Twelve included studies (11 case series and 1 RCT) met the eligibility criteria. The length of the follow‐up ranged from 1 week to 2 years after surgery. The majority of studies were carried out in Asia. Vitrectomy showed improvement in BCVA from baseline. The studies exhibited a wide range in the mean change of BCVA, with the greatest median change 12 months after surgery (0.29, IQR: 0.065). The RCT showed no significant difference between surgery and watchful waiting. Three studies used the National Eye Institute 25‐Item Visual Function Questionnaire (NEI‐VFQ‐25) as their only patient‐reported outcome to measure vision‐related quality of life for iERM. The composite scores of NEI‐VFQ‐25 were significantly improved at 3 and 12 months after surgery. Vitrectomy surgery was found to be cost‐effective in one included study, with an incremental cost‐effectiveness ratio of $4680 per quality‐adjusted life year gained.

**Conclusion:**

Vitrectomy surgery demonstrated improvement in BCVA from baseline, but the RCT with a watchful waiting group showed no significant difference. Vitrectomy surgery was cost‐effective. An RCT comparing iERM vitrectomy surgery and either delayed surgery or no surgery, and a health economics evaluation of the intervention are needed to confirm the effectiveness and cost‐effectiveness of iERM vitrectomy surgery.

## 1. Introduction

Primary idiopathic epiretinal membrane (iERM), or premacular membrane, is a common finding that is present in up to 34% of people aged 60 or over [1, 2]. iERM refers to a thin layer of preretinal scar tissue or membrane over the macula in the absence of other eye disease [3]. It can be asymptomatic in the early stages and does not always progress [1]. If it does progress and cause tractional changes to the central macula, people may experience distorted and blurred vision, finding difficulty especially in reading and seeing detail [4]. The condition mostly affects one eye; however, about 10% of iERM may affect both eyes with differing severity [1]. The condition does not affect peripheral vision; hence, it does not lead to total loss of sight or blindness [1]. Clinical examinations by fundal photography and ocular coherence tomography (OCT) are used for the diagnosis [3].

When there are symptoms affecting activities of daily living, vitrectomy and membrane peeling surgery (hereon referred to as ‘surgery’) are commonly conducted [3]. The surgery aims to remove the membrane and improve the traction‐induced visual disturbance [3, 5]. The surgery is typically performed under a local anaesthetic and usually takes up to 1 hour. After surgery, patients receive a short course of eye drops to prevent infection and aid ocular recovery. However, there are currently no standardised or accepted criteria for performing surgery for iERM [6–8]. The optimum timing of surgery is also currently undefined [3]. Therefore, indications for conducting the surgery are based on patients’ symptoms and clinicians’ judgements, with variable thresholds from country to country, surgeon to surgeon and patient to patient [5].

The outcomes of surgery are variable. Positive outcomes of surgery can be seen within three to 6 months postoperatively in 70%–80% of patients [9], but 30% of patients do not notice a significant improvement, and 5% report worse vision after surgery [10, 11]. Depending on the study, up to 20% of patients will require further surgery [10]. In one RCT, surgery showed an improvement in BCVA compared to the watchful waiting/deferred surgery [12]. However, no significant difference was found, and the study was judged as showing a low certainty of evidence [12]. To date, there are no RCTs directly investigating the effect of vitrectomy compared to no intervention in people with iERM [12]. Additionally, there is little cost‐effectiveness evidence for iERM surgery [13]. The direct cost of vitrectomy surgery in the United Kingdom in 2015 was estimated at around £1,700, including staff costs (£297), consumables (£619), equipment (£82), out‐of‐surgery costs (£260) and overheads (30%) [14].

Due to the uncertainties regarding outcomes and cost‐effectiveness of surgery, this review focussed on two components of clinical effectiveness and cost‐effectiveness. This review aimed to assess the effectiveness and cost‐effectiveness of surgery, including how existing studies measured effectiveness, the patient‐reported outcome measures (PROMs) used for both effectiveness and health economic analysis, and whether there was any patient and public involvement (PPI) in the studies.

The overarching question this systematic review aims to answer is:

What is the effectiveness and cost‐effectiveness of surgery on iERMs?

The sub‐questions were:

a. What outcomes/PROMs were used to measure effectiveness?
b. What outcomes/PROMs were used for the economic analysis?
c. Was there any PPI in the study?

## 2. Materials and Methods

The review protocol was registered before the searches in PROSPERO (CRD42024542913) [15]. The systematic review adhered to the Preferred Reporting Items for Systematic Reviews and Meta‐Analyses (PRISMA) checklist and the Synthesis Without Meta‐analysis (SWiM) reporting guidelines. The search strategy was adapted from a current systematic review [11], ensuring a valid and comprehensive approach (Supporting Appendix 1). The date of the search was 10 May 2024.

### 2.1. Eligibility Criteria

The eligibility criteria were formulated based on the population, intervention, comparator and outcomes (PICO) framework (Supporting Appendix 1). Studies were included in the systematic review if they met the following criteria: (1) The population were adults over the age of 18 years with symptomatic iERMs causing macular pucker of any type. (2) Prospective studies for the effectiveness of iERM surgery and health economic studies for the cost‐effectiveness of iERM surgery. (3) Intervention was vitrectomy. (4) Articles published from 1st January 2000 to 1st May 2024 in the English language. (5) The article was available as a full text. Systematic reviews, scoping reviews, rapid reviews, editorials, letters to editors, conference abstracts, commentaries and viewpoint articles were excluded. When an article was unavailable as a full text, direct contact with the authors was made.

### 2.2. Study Selection and Data Extraction

Relevant reviews were selected according to the eligibility criteria using a two‐step screening process, first screening titles and abstracts and second screening full text. Two independent reviewers (KD and HTK) performed screening, study selection and data extraction. Disagreements were resolved by the third researcher (TS or RTE). Rayyan reviewing software was used [16]. The reviewers extracted data from the included studies using a predefined data extraction form on general characteristics of studies, descriptive characteristics of studies, public and patient involvement, PROMs and key findings.

### 2.3. Quality Assessment

Two reviewers (KD and HK) assessed the quality of included studies. The JBI’s critical appraisal tools were used for quality assessment of included studies (case series and RCTs) [17, 18]. The risk of bias of the RCT study was assessed by the risk‐of‐bias 2 tool [19]. The Consolidated Health Economic Evaluation Reporting Standards 2022 (CHEERS 2022) Statement was also used for the included health economics study [20]. Discrepancies were resolved through discussion with a third reviewer.

### 2.4. SWiM

The primary outcome of the effectiveness of vitrectomy surgery for iERMs was reported in 9 studies by mean best‐corrected visual acuity (BCVA) (logMAR) (8 case series, 1 RCT) and by median (interquartile range/range) in 3 case series studies [21–23]. Due to a lack of individual patient‐level data, converting median to mean was not possible. In addition, the main outcomes were reported at multiple and different follow‐up periods. Therefore, meta‐analysis could not be carried out. SWiM was applied following the SWiM in the systematic review reporting guideline [24], where the same types of study reported the same outcomes at the same follow‐up period. The summarising effect estimates method was used to synthesise the mean change of BCVA after surgery to assess what the range and distribution of observed effects were. Thus, the results from 8 case series studies were analysed. *R* software and Microsoft Excel were used to conduct this analysis. A box‐and‐whisker plot was used to present the synthesis results as recommended by Cochrane [25]. Sensitivity analysis was also conducted to examine the impact of the quality of included studies.

## 3. Results

For the effectiveness of vitrectomy surgery of iERMs, 3011 studies were identified. 691 of 3011 studies were removed due to duplication. 2320 studies were included for title and abstract screening. 16 studies were assessed for eligibility through full‐text screening. Four studies were excluded due to duplication [26], no observed BCVA at the follow‐up period [27], and lack of type of iERM [28, 29]. Therefore, 12 studies were included in the review of the effectiveness of vitrectomy surgery for iERMs, of which 11 studies were case series studies and one was an RCT.

Additionally, for the cost‐effectiveness of vitrectomy surgery of iERMs, 164 studies were identified from four databases (Embase, Medline, Scopus and Cochrane Library). Of these, 29 of 164 were removed due to duplication. One study was eligible and included [13]. In addition, 514 studies were identified from two grey literature databases (Google and Semantic Scholar). None were eligible for screening. The PRISMA flow charts of the study inclusion are presented in Supporting Appendix 2.

### 3.1. The Effectiveness of Vitrectomy Surgery for iERMs

**Figure 1:**
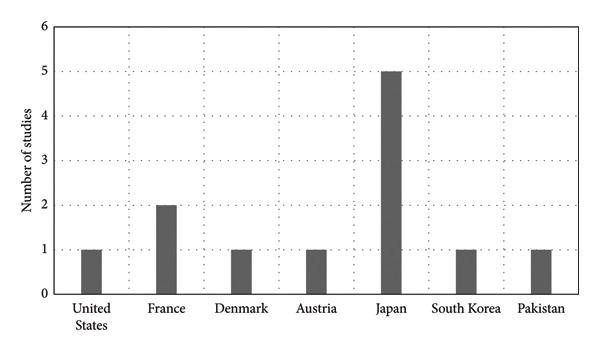
Distribution of countries of included studies.

The most common type of vitrectomy performed was 23‐gauge vitrectomy, which was performed in five studies. Out of 12 studies, seven (58%) were conducted in Asia, five of which in Japan [22, 30–33], as illustrated in Figure 1. The number of participants ranged from 10 to 53, with mean age ranging from 63 to 72 years. One eye of participants was examined in 11 studies, but the study by Mieno et al. examined both eyes [22]. From the 12 studies, two (17%) had comparator groups (one age‐matched normal control and one watchful waiting group) [26, 30] where the age‐matched normal control was compared with the intervention group in terms of vision‐related function, measured by the NEI‐VFQ‐25 only [30, 34]. The duration of follow‐up varied from 1 week to 2 years after surgery. Other details of the included studies are presented in Table 1.

**Table 1:** Baseline characteristics of the included studies (*n* = 12).

Study ID	Author	Year	Country	Study design	Intervention	Comparator	Number of participants	Mean age (SD)	Sex (male%)	Follow‐up time (months)
1	Tari et al. [35]	2007	United States	Case series	Three‐port vitrectomy^β^	No	10	63.9 (9.5)	60%	3
2	Schweitzer et al. [36]	2009	France	Case series	23‐Gauge	No	26	71.7 (8.7)	65%	1
3	Okamoto et al. [30]	2009	Japan	Case series	20‐Gauge	Age‐matched normal control^α^	28	66.7 (8.5)	46%	3
4	Kinoshita et al. [37]	2012	Japan	Case series	25‐Gauge	No	49	70.2 (1.2)	43%	1 and 12
5	Matsuoka et al. [32]	2012	Japan	Case series	25‐Gauge	No	26	70 (9)	38%	3 and 12
6	Kim et al. [38]	2013	South Korea	Case series	23‐Gauge	No	52	62.1 (7.5)	46%	2 and 6
7	Kofod et al. [26]	2016	Denmark	RCT	23‐Gauge	Watchful waiting	53 (20 for surgery, 33 for watchful waiting)	Surgery: 69 (3) Watchful waiting: 66 (3)	Surgery: 35% Watchful waiting: 48%	1, 3, 6, 9 and 12
8	Shahzadi et al. [21]	2016	Pakistan	Case series	23‐Gauge	No	30	72 (5)	43%	6
9	Takabatake et al. [33]	2018	Japan	Case series	Small‐gauge^β^	No	45	64.8 (8.6)	36%	6 and 12
10	Mieno et al. [22]	2020	Japan	Case series	25‐ Or 27‐gauge	No	42	69 (2.5)	42%	3,6 and 12
11	Khanna et al. [23]	2022	France	Case series	25‐Gauge	No	42	72.7 (7.4)	58%	6 and 24
12	Hollaus et al. [39]	2023	Austria	Case series	23‐Gauge	No	48	70 (6.7)	50%	1 week, 1 and 3 months

^β^Data on the gauge were unavailable.

^α^Age‐matched normal control was used for comparison with the intervention group in terms of vision‐related function measured by the NEI‐VFQ‐25 only.

Two included studies had comparison groups (one RCT and one case series). The results from the RCT demonstrated that participants from both surgery and watchful waiting groups gained significant improvement of BCVA at 12‐month follow‐up (surgery group: *p* < 0.001, watchful waiting: *p* = 0.03). Due to data availability, the mean change of BCVA from baseline was assumed to be 5 and 3 Early Treatment of Diabetic Retinopathy Study (ETDRS) letters for the surgery and watchful waiting groups, respectively. These results can be converted to logMAR as 1.60 and 1.64, respectively. However, the change of BCVA after surgery was not significantly different between the two groups (*p* = 0.30) [26]. Another case series study compared the vision‐related quality of life (VRQoL) between the intervention and age‐matched control using the NEI‐VFQ‐25. There was no comparison between the intervention group and the age‐matched normal control in terms of BCVA after surgery [30].

PROMs were used to capture VRQoL in three studies (25%) [23, 30, 32]. The NEI‐VFQ‐25 was the only PROMs that was used [34]. Vitrectomy caused significant improvement of the composite scores of NEI‐VFQ‐25 at 3 months [30] and 12 months after surgery [23, 30, 32]. Vitrectomy also caused significant improvement in three subscales’ scores of the NEI‐VFQ‐25 (general vision, mental health and driving) at 2 years after surgery, in comparison to baseline [23]. In contrast, participants with iERM had significantly lower pre‐ and postoperative NEI‐VFQ‐25 composite scores than participants in the age‐matched normal control group (*p* < 0.0001) [30]. The postoperative NEI‐VFQ‐25 composite score demonstrated significant correlation with the severity of metamorphopsia [30] and BCVA (logMAR) at postoperation [30, 32].

Three studies (25%) reported the main primary outcome as median BCVA [21–23]. The median change from baseline and follow‐up cannot be calculated. Vitrectomy surgery caused significant improvement in BCVA at 3, 6, 12 and 24 months after surgery compared with baseline (Supporting Appendix 3).

Five studies (42%) reported complications of surgery [21, 26, 30, 32, 36], of which four reported that no significant complications occurred during or after surgery [26, 30, 32, 36]. In one study, recurrence of iERM was found to be the most frequent complication, with four out of 30 participants (13.3%) experiencing this complication [21]. These recurrences did not affect BCVA, and none of the participants underwent repeat surgery [21]. Additionally, 3.3% of participants had retinal detachment at 1 month after surgery. These participants underwent repeat surgery with no improvement of BCVA [21].

None of the included studies (*n* = 12) reported PPI. Data extraction of included studies of the effectiveness of vitrectomy surgery for iERMs are provided in Supporting Appendix 4.

### 3.2. Quality Assessment

The quality of the included studies was evaluated using the JBI’s critical appraisal tools (case series and randomised controlled trials [RCTs]) [17, 18]. Risk of bias of the included RCT was also assessed by using the risk of bias 2 tool [19]. However, the risk of bias in case series studies was not assessed, as this study type inherently carries a high risk of bias.

Three studies (25%) were judged as being moderate in quality [26, 35, 39], and the other 9 studies were judged as high quality (Table 2). The study by Kofod et al. (2015) was the only RCT included and had some concern about risk of bias [26]. Details of the quality assessment and risk of bias of the included studies are provided in Supporting Appendix 5.

**Table 2:** Quality assessment of the included studies (*n* = 12).

Study ID	Author	Study design	Number of items reported as yes	Quality of study
1	Tari et al. [35]	Case series	6 out of 10	Moderate
2	Schweitzer et al. [36]	Case series	10 out of 10	High
3	Okamoto et al. [30]	Case series	8 out of 10	High
4	Kinoshita et al. [37]	Case series	8 out of 10	High
5	Matsuoka et al. [32]	Case series	10 out of 10	High
6	Kim et al. [38]	Case series	8 out of 10	High
7	Kofod et al. [26]	RCT	9 out of 13	Moderate
8	Shahzadi et al. [21]	Case series	9 out of 10	High
9	Takabatake et al. [33]	Case series	9 out of 10	High
10	Mieno et al. [22]	Case series	10 out of 10	High
11	Khanna et al. [23]	Case series	9 out of 10	High
12	Hollaus et al. [39]	Case series	7 out of 10	Moderate

### 3.3. SWiM

The 8 case series studies [30, 32, 33, 35–39] were grouped to conduct SWiM due to data availability. These studies reported the mean BCVA at baseline and follow‐up at overlapped follow‐up time points. One study reported the mean BCVA as the ETDRS letters; hence, the results were converted to logMAR using a recognised conversion method [40]. The mean change of BCVA from baseline to follow‐up at each timepoint was calculated and presented with standard deviation (SD). The mean change of BCVA at each follow‐up period was obtained by subtracting the follow‐up mean BCVA from the baseline mean BCVA. Data were not sufficient to calculate the SD of the changes (data of the mean and SD change were unavailable in all included studies). Imputing a change‐from‐baseline SD was conducted using a correlation coefficient equation (assuming a correlation coefficient of 0.5 [41]) [25]. Seven out of 8 studies (88%) included in the data synthesis were judged as high quality except the study by Hollaus et al. (2023) (moderate) [39].

**Figure 2:**
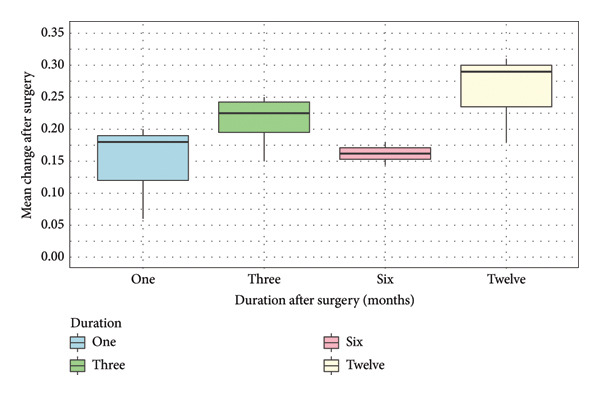
Box‐and‐whisker plot of mean change after surgery over 12 months.

The mean change of BCVA from baseline to follow‐up time points was summarised (Table 3). The results are also presented in a box‐and‐whisker plot (Figure 2) as recommended by Cochrane [25]. The median changes of BCVA (IQR) at 1, 3, 6 and 12 months after surgery were 0.18 (0.07), 0.23 (0.048), 0.16 (0.018) and 0.29 (0.065), respectively. The greatest median change of BCVA was shown 12 months after surgery (0.29, IQR: 0.065), while the lowest median change of BCVA was shown at 6 months after surgery (0.16, IQR: 0.018) (Figure 2).

**Table 3:** Mean BCVA change after iERM surgery over 12 months.

Study/Author/Year	Mean BCVA (logMAR)		Mean change (SD)
	Baseline	Follow‐up point	
*1 month*			
Schweitzer et al. [36]	0.38 (0.03)	0.20 (0.03)	0.18 (0.030)
Kinoshita et al. [31]	0.53 (0.27)	0.33 (0.25)	0.20 (0.258)
Hollaus et al. [39]	0.30 (0.24)	0.24 (0.17)	0.06 (0.214)

*3 months*			
Tari et al. [35]	0.40 (0.11)	0.19 (0.13)	0.21 (0.121)
Okamoto et al. [30]	0.495 (0.293)	0.245 (0.294)	0.25 (0.294)
Matsuoka et al. [32]	0.41 (0.05)	0.17 (0.04)	0.24 (0.046)
Hollaus et al. [39]	0.30 (0.24)	0.15 (0.16)	0.15 (0.212)

*6 months*			
Kim et al.^β^ [38]	0.32 (0.17)	0.18 (0.14)	0.14 (0.159)
Takabatake et al. [33]	0.17 (0.05)	−0.01 (0.04)	0.18 (0.045)

*12 months*			
Schweitzer et al. [36]	0.38 (0.03)	0.09 (0.03)	0.29 (0.030)
Matsuoka et al. [32]	0.41 (0.05)	0.10 (0.03)	0.31 (0.044)
Takabatake et al. [33]	0.17 (0.05)	−0.01 (0.04)	0.18 (0.045)

^β^BCVA was reported as ETDRS letters; the results were converted to logMAR using a recognised conversion method [40].

### 3.4. Sensitivity Analysis

**Figure 3:**
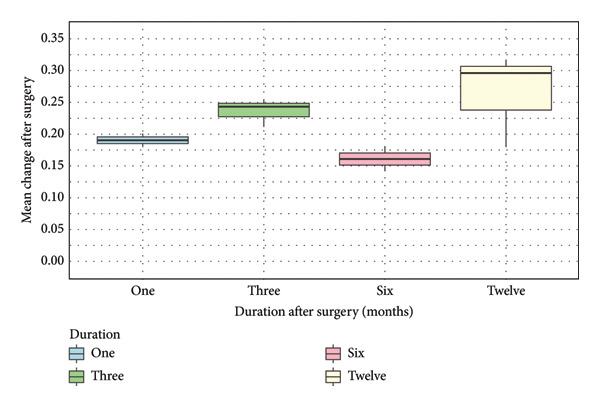
Box‐and‐whisker plot of mean change after surgery over 12 months (sensitivity analysis).

Only one study by Hollaus et al. (2023) was assessed as being of moderate quality. We conducted sensitivity analysis by excluding the results from Hollaus et al. (2023). The median change of BCVA (IQR) at 1 month after surgery changed from 0.18 (0.070) to 0.19 (0.010). Additionally, the median change of BCVA at 3 months after surgery was from 0.23 (0.048) to 0.24 (0.020) (Figure 3).

### 3.5. The Cost‐Effectiveness of Vitrectomy Surgery of iERMs

Only one study by Gupta et al. (2008) was eligible for this review in terms of the cost‐effectiveness of vitrectomy surgery for iERMs. This study performed a computer‐based, value‐based medicine analysis and cost‐utility analysis [13]. The data used were obtained from two large cohort studies, namely the Blue Mountain Eye and Beaver Dam Eye Studies [42, 43]. The Blue Mountain Eye Study was carried out in Australia and included data on the 5‐year cumulative incidence and change in iERM in the older Australian population. This population was representative of Australia in terms of income and education [42]. The Beaver Dam Eye Study was conducted in the United States and aimed to describe the change in BCVA over a 10‐year period in participants who lived in the city or township of Beaver Dam, aged 43–84 years at the time of recruitment [43]. For BCVA and complications outcomes, the BCVAs and complications following surgery data were obtained from another three studies of 90 eyes in total that had undergone surgery [44–46].

Decision analyses were performed using a decision tree and Markov modelling. A decision tree analysis was performed for iERM surgery in the better‐seeing eye (with the assumption that the surgery was definitive). Markov model analysis was conducted for iERM surgery in the worse‐seeing eye scenario, capturing the risk of recurrent iERM. Utility values were obtained from converting BCVAs using a time trade‐off utility, following the research by Brown (1999) [47]. The cost‐utility analysis was conducted from a third‐party insurer cost perspective, including only direct medical costs. A discount rate of 3% was applied for costs and utilities.

The results of Gupta et al. (2008) demonstrated that iERM surgery was substantially cost‐effective. The incremental cost‐effectiveness ratio (ICER) of iERM surgery in the better‐seeing eye compared with observation was $4680 per QALY (the willingness‐to‐pay threshold of the United States is between $50,000 and $100,000 per QALY) [48]. The ICER of iERM in the worse‐seeing eye compared with observation was $16,146 per QALY.

Data from PPI were not reported (Table 4). This study was judged as being moderate to high quality (Supporting Appendix 6). Following the CHEERS 2022 checklist, three items were not reported (health economic analysis plan, approach to engagement with patients and others affected by the study, and effect of engagement with patients and others affected by the study) (Supporting Appendix 6).

**Table 4:** Data extraction of included studies for the cost‐effectiveness of vitrectomy surgery for iERMs.

Author, Year, Country	Gupta et al., 2008, United States
Study objective	To evaluate the iERM surgery in the better‐ and the worse‐seeing eye and determine the cost‐effectiveness of this procedure
Type of study	A computer‐based, value‐based medicine and cost‐utility analysis
Population	This study used the data from two published studies (the Blue Mountain Eye Study [Australian] and the Beaver Dam Eye Study [United States]).
	• Blue Mountain Eye Study (*n* = 3654) [42]
	o Participants were 49 years or older
	• Beaver Dam Eye Study (*n* = 3684) [43]
	o Participants were 43–86 years
Intervention	iERM surgery
Outcomes	Direct medical costs and utility (quality‐adjusted life year, QALY)
PPI involved?	Not reported
PROMs measures?	No. The utility values were obtained from the conversion of visual acuity by using a modified VF‐14 questionnaire
Summary	iERM surgery was substantially cost‐effective. The incremental cost‐effectiveness ratio (ICER) of iERM surgery in the better‐seeing eye compared with observation is $4680 per QALY (the cost‐effective threshold is $50,000 per QALY). The ICER of iERM in the worse‐seeing eye compared with observation is $16,146 per QALY.

## 4. Discussion

This systematic review evaluated the visual effectiveness and cost‐effectiveness of surgery for iERMs. Only one RCT and 11 case series studies were included in terms of the effectiveness of surgery. No additional RCTs were found from an existing systematic review of surgery for iERM, which included RCTs and did not include cost‐effectiveness studies [11]. The results from 12 included studies demonstrated that surgery caused a significant improvement in iERM, measured with BCVA compared with baseline BCVA. However, the improvement of BCVA was not significantly different when compared with a watchful waiting group [26]. It is possible that these results do not fully represent watchful waiting, as 8 participants crossed over from the watchful waiting group to surgery, which affected the gain of BCVA after surgery [26]. To the author’s knowledge, there are no RCTs that directly assess the effectiveness of vitrectomy surgery for iERMs compared with no intervention.

The 8 case series studies were grouped to conduct data synthesis using summarising effect estimates. There is a possible trend of an improvement of mean change of BCVA from 1 to 12 months after surgery, except at 6 months (Figure 2). A possible explanation for this might be the better mean preoperative BCVA from the results conducted by Takabatake et al. (2018) [33]. Participants in this study had mild severity of iERM, and 87% of participants (*n* = 39) received combined cataract extraction and surgery, which could potentially affect the effectiveness of the surgery in terms of visual improvement [33]. The sensitivity analysis using only the high‐quality studies showed a greater improvement of median change of BCVA at 1 and 3 months after surgery (Figure 3).

Only five out of 12 included studies captured complications following surgery. Of these, four studies reported no significant complications during and after surgery [26, 30, 32, 36]. Recurrence of iERM (13.3%) and retinal detachment (3.3%) after surgery were reported by one study [21]. Compared to other studies, the postoperative retinal detachment rate was higher than previously reported [49], and the recurrence rate of iERM was lower (13.3% vs. 39.5%) [50]. However, observed recurrence rates of iERM will vary depending on the definition of recurrence used and other surgical variables, including whether ILM peeling was carried out [50, 51]. Additionally, the reporting of complications in the included studies was not systematic, as they primarily focussed on the effectiveness of the surgery, and furthermore, they were all small, reducing the reliability of detecting rare complications such as retinal detachment. Therefore, these findings likely under‐represent true complication rates and should be interpreted with caution.

Only one study of cost‐utility analysis of surgery for iERMs was found [13]. Surgery for iERMs was cost‐effective when compared with observation from a third‐party insurer cost perspective. It was carried out using participants’ data from two large cohort studies [42, 43]. BCVA outcomes and complications were derived from three other studies [44–46]. Utilities were calculated by converting BCVAs using Brown’s equation (47). However, none of the participants with iERMs were included in the study that informed Brown’s equation. The accuracy and reliability of this equation may not reflect the utility values of participants with iERM. In addition, value sets are different depending on various factors, such as country, healthcare system and level of development of the country, religion and language [52]. Furthermore, determining which intervention is cost‐effective requires considering the health system, perspective, and the willingness‐to‐pay threshold [53], which can vary from country to country. Therefore, these results should be interpreted with caution.

The NEI‐VFQ‐25 was the only PROM used in the included studies. The NEI‐VFQ‐25 is a visual function questionnaire (25‐item) measuring the impact of visual impairment on quality of life [34] and is commonly employed in ophthalmology research [54]. The composite score of NEI‐VFQ‐25 and subscale scores for each domain can be reported, ranging from 0 to 100 (a higher score represents better visual function) [34]. The subscales of NEI‐VFQ‐25 can be used to estimate the EQ‐5D‐based utility scores [55]. This systematic review found that vitrectomy caused significant improvement in VRQoL at 3 months and 12 months after surgery [23, 30, 32].

To the author’s knowledge, there is no study that directly measured the generic health‐related quality of life (HRQoL) of participants with iERMs. The EuroQoL is a generic HRQoL questionnaire commonly used in health economics [52]. It allows the calculation of utilities and has been recommended for cost‐utility analyses by the National Institute for Health and Care Excellence [56]. A further health economics study of the cost‐effectiveness of surgery for iERM is needed to explore the quality of life of people with iERMs using both generic and VRQoL questionnaires (the EuroQol and NEI‐VFQ‐25).

This review has several strengths. We adapted the search strategy from a current systematic review [11], ensuring a valid and comprehensive search strategy. This review also adhered to the PRISMA and SWiM guidelines. However, limitations to this review include heterogeneity in reporting outcomes and types of study included. The 8 case series grouped for data synthesis were mostly carried out in Asia and were lacking control groups, potentially impacting the generalisability of the findings. Additionally, the main outcomes were reported at multiple and different time points, and few studies were able to synthesise the outcomes. This review’s findings were therefore limited by the small number of eligible studies at each time point.

This review highlights a paucity of data on the impact of vitrectomy for iERM. Due to the uncertainty surrounding the effectiveness and cost‐effectiveness of iERM surgery, a future RCT and a health economic analysis are recommended. These studies should aim to investigate both the visual effectiveness and cost‐effectiveness of iERM surgery. The mean BCVA measured using the logMAR scale should be designated as the primary outcome for assessing effectiveness. Consideration should be given to including other measures of visual function, including assessment of metamorphopsia and aniseikonia, important and frequent symptoms in patients with iERM. To evaluate patients’ quality of life, both generic and VRQoL outcomes should be examined using the EuroQol (EQ‐5D) and the NEI‐VFQ‐25. The research would also assist in the development of standardised guidelines for vitrectomy in people with iERM to ensure future consistency in clinical practice.

## 5. Conclusion

Vitrectomy surgery improves BCVA (logMAR) in people with iERMs, but there is uncertainty as to the benefits when compared with people with iERMs of similar stages who do not undergo surgery. Vitrectomy surgery is cost‐effective, as informed by data from cohort studies. A RCT and health economics evaluation of iERM vitrectomy surgery are needed to confirm the effectiveness and cost‐effectiveness of iERM surgery and should include strong PPI.

## Ethics Statement

The authors have nothing to report.

## Consent

The authors have nothing to report.

## Disclosure

All authors approved the final manuscript.

## Conflicts of Interest

The authors declare no conflicts of interest.

## Author Contributions

K.D., T.S., D.H.S., N.H. and R.T.E. participated in the design of the structure of the research manuscript. K.D. and H.T.K. contributed to the review of the systematic review and managed data collection. K.D. conducted analysis. T.S. and D.H.S. provided clinical expertise and were involved with the manuscript development. N.H. and R.T.E. supervised and were involved with the manuscript development. All authors reviewed the final manuscript.

## Funding

The authors received no specific funding for this work.

## Supporting Information

Additional supporting information can be found online in the Supporting Information section.

## Supporting information


**Supporting Information 1** Appendix file 1: Search strategy.

**Supporting Information 2** Appendix file 2: PRISMA flow chart.

**Supporting Information 3** Appendix file 3: Median BCVA after surgery.

**Supporting Information 4** Appendix file 4: Data extraction of included studies for the effectiveness of vitrectomy surgery for iERM.

**Supporting Information 5** Appendix file 5: Quality and risk of bias assessment of included studies for the effectiveness of vitrectomy surgery for iERM.

**Supporting Information 6** Appendix file 6: Quality assessment for the included study for the cost‐effectiveness of vitrectomy surgery for iERM.

**Supporting Information 7** Appendix file 7: PRISMA 2020 abstract checklist.

**Supporting Information 8** Appendix file 8: PRISMA 2020 checklist.

**Supporting Information 9** Appendix file 9: SWiM reporting item checklist.

## Data Availability

All data generated or analysed during this study are included in this published article and its supporting information files.
