# Supplementary material for: The Visual Effectiveness and Cost‐Effectiveness of Vitrectomy and Membrane Peeling for Primary Idiopathic Epiretinal Membranes (iERMs): A Systematic Review
Source: J Ophthalmol. 2026 Jan 4;2026:5546933. doi: 10.1155/joph/5546933 (PMC12767011; doi:10.1155/joph/5546933)
Supplement: Supplementary file 1 — Supporting Information 1 Appendix file 1: Search strategy. [file JOPH-2026-5546933-s008.docx]

## **Appendix 1: Search strategy**

From the research questions, a PICO search strategy was applied (Table 1), and search terms were selected and adapted from the published systematic review by Yusuf et al. (2019) [1]. Figures 1 and 2 demonstrate the search terms for Medline via Ovid database. Figure 3 illustrates the search terms for Google and Semantic Scholar.

### **Table 1: Application of PICO on inclusion criteria and search strategy**

| **PICO** | **Inclusion criteria** | **Search terms** |
| --- | --- | --- |
| Population | Adults with symptomatic idiopathic epiretinal membranes causing macular pucker of any type. | ‘Epiretinal membrane’ or ‘macular pucker’ |
| Intervention | surgery or pars plana vitrectomy or membrane peeling | ‘Vitrectomy’ or ‘PPV’ or ‘membrane peeling’ |
| Comparison | N/A | N/A |
| Outcome | The main outcome of effectiveness of vitrectomy surgery will be the change in visual acuity in the eye that received surgery from baseline (pre-surgery) to follow-up (post-surgery). This will be assessed using the Early Treatment Diabetic Retinopathy Study (ETDRS) or other measurement tools.  The main outcomes of the cost-effectiveness studies will depend on the types of health economics studies found in the literature search (e.g., the incremental cost-effectiveness ratio (ICER), social return on investment ratio, cost per extra ETDRS).  In addition, patient-reported outcome measures (PROMs) will be identified in both the effectiveness and cost-effectiveness studies (e.g., the EuroQoL-5D questionnaire). | Type of included studies will be used as search terms. For example, ‘randomised controlled trial’ or ‘cost-benefit analysis’ |

### **Figure 1. Search terms for the effectiveness of operating primary idiopathic epiretinal membranes**

1 Epiretinal Membrane/

2 (epiretinal adj2 membrane$).tw.

3 ERM.tw.

4 (membrane$ adj2 (epimacular or premacular or preretinal)).tw.

5 (cellophane adj2 (maculopath$ or retinopath$)).tw.

6 premacular fibrosis.tw.

7 macular pucker$.tw.

8 ((retina$ or retinopath$) adj2 wrinkl$).tw.

9 1 or 2 or 3 or 4 or 5 or 6 or 7 or 8

10 exp Vitrectomy/

11 vitrectom$.tw.

12 PPV.tw.

13 ((ILM or membrane) adj2 peel$).tw.

14 (foveal adj2 spar$).tw.

15 10 or 11 or 12 or 13 or 14

16 9 and 15

17 limit 16 to (english language and yr="2000 - 2024")

18 limit 17 to humans

19 randomised controlled trial.pt.

20 (randomised or randomized).ab,pt,ti.

21 trial.ab,ti.

22 randomly.ab,ti.

23 prospective.tw.

24 19 or 20 or 21 or 22 or 23

25 18 and 24

### **Figure 2** **Search terms for the cost effectiveness of operating primary idiopathic epiretinal membranes**

1 Epiretinal Membrane/

2 (epiretinal adj2 membrane$).tw.

3 ERM.tw.

4 (membrane$ adj2 (epimacular or premacular or preretinal)).tw.

5 (cellophane adj2 (maculopath$ or retinopath$)).tw.

6 premacular fibrosis.tw.

7 macular pucker$.tw.

8 ((retina$ or retinopath$) adj2 wrinkl$).tw.

9 1 or 2 or 3 or 4 or 5 or 6 or 7 or 8

10 exp Vitrectomy/

11 vitrectom$.tw.

12 PPV.tw.

13 ((ILM or membrane) adj2 peel$).tw.

14 (foveal adj2 spar$).tw.

15 10 or 11 or 12 or 13 or 14

16 9 and 15

17 health economic$.mp.

18 exp Cost-Benefit Analysis/

19 cost effective$.tw.

20 cost-effectiveness.tw.

21 cost-utility.tw.

22 exp "Cost of Illness"/ or exp "Costs and Cost Analysis"/

23 cost consequence.tw.

24 social return on investment.tw.

25 budget impact analysis.tw.

26 markov model.tw.

27 decision analytic modelling.tw.

28 economic evaluation.tw.

29 17 or 18 or 19 or 20 or 21 or 22 or 23 or 24 or 25 or 26 or 27 or 28

30 16 and 29

31 limit 30 to (english language and yr="2000 - 2024")

### **Figure 3 Search terms for grey literature for the cost effectiveness of operating primary idiopathic epiretinal membranes**

Google Search Engine

| **#** | **Search** |
| --- | --- |
| 1 | "cost effectiveness" OR "health economic*" AND "idiopathic epiretinal membrane" AND vitrectomy |
| 2 | "cost effectiveness" OR "health economic*" AND "macular pucker" AND vitrectomy |
| 3 | "cost effectiveness" OR "health economic*" AND "macular pucker" AND "pars plana" |
| 4 | "cost effectiveness" OR "health economic*" AND "idiopathic epiretinal membrane" AND “pars plana” |

Semantic Scholar Search Engine

| **#** | **Search** |
| --- | --- |
| 1 | cost effectiveness OR health economic* AND idiopathic epiretinal membrane AND vitrectomy |
| 2 | cost effectiveness OR health economic* AND macular pucker AND vitrectomy |
| 3 | cost effectiveness OR health economic* AND macular pucker AND pars plana |
| 4 | cost effectiveness OR health economic* AND idiopathic epiretinal membrane AND pars plana |

**References**

[1] Yusuf A, Bizrah M, Bunce C, Bainbridge JW. Surgery for idiopathic epiretinal membrane. Cochrane Database of Systematic Reviews 2019;2019. https://doi.org/10.1002/14651858.CD013297.
