# Supplementary material for: The Visual Effectiveness and Cost‐Effectiveness of Vitrectomy and Membrane Peeling for Primary Idiopathic Epiretinal Membranes (iERMs): A Systematic Review
Source: J Ophthalmol. 2026 Jan 4;2026:5546933. doi: 10.1155/joph/5546933 (PMC12767011; doi:10.1155/joph/5546933)
Supplement: Supplementary file 2 — Supporting Information 2 Appendix file 2: PRISMA flow chart. [file JOPH-2026-5546933-s001.pdf]

Appendix 2. The PRISMA flow charts of the effectiveness and cost-effectiveness of vitrectomy surgery for iERMs

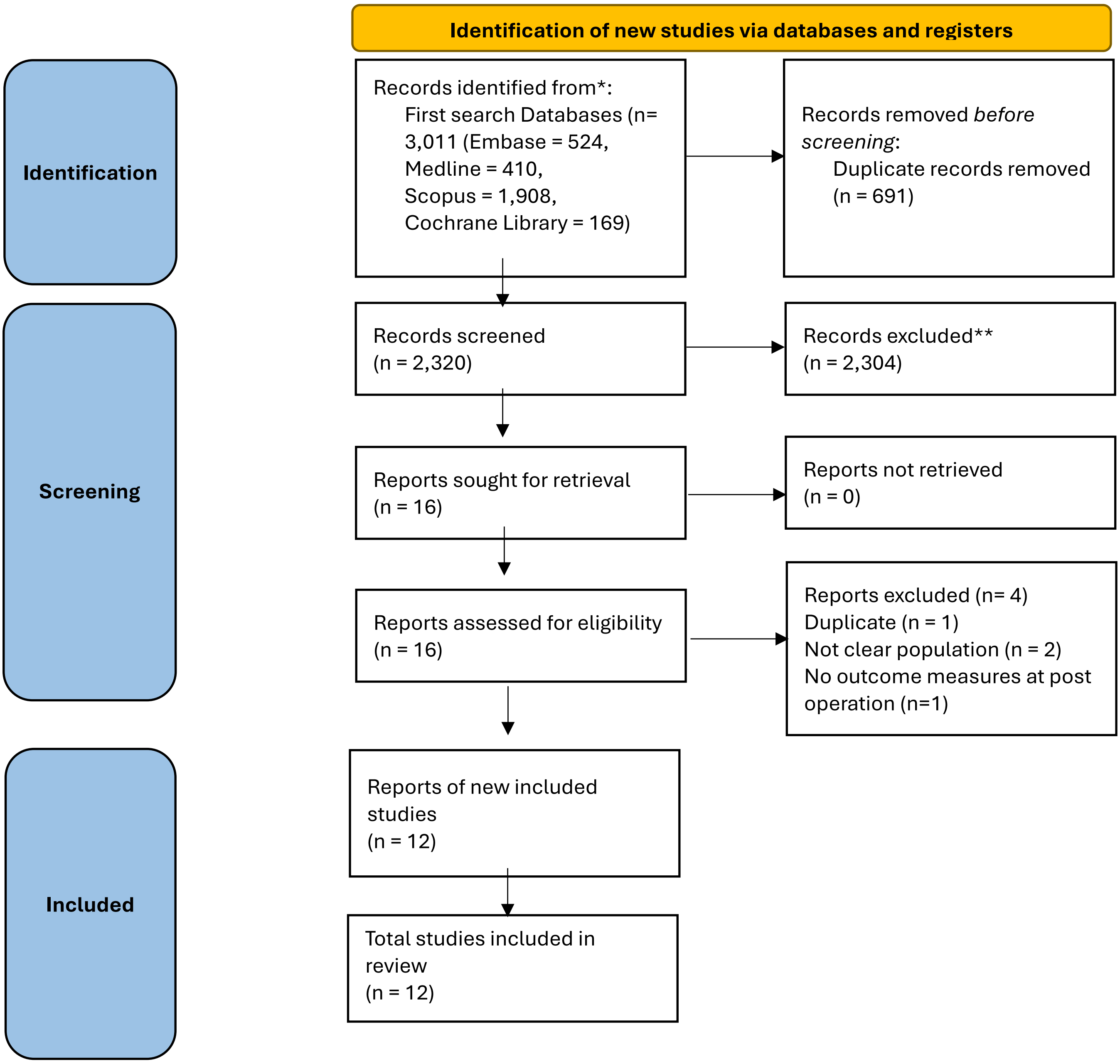

Figure 1. The PRISMA flow chart of the effectiveness of vitrectomy surgery for primary iERMs

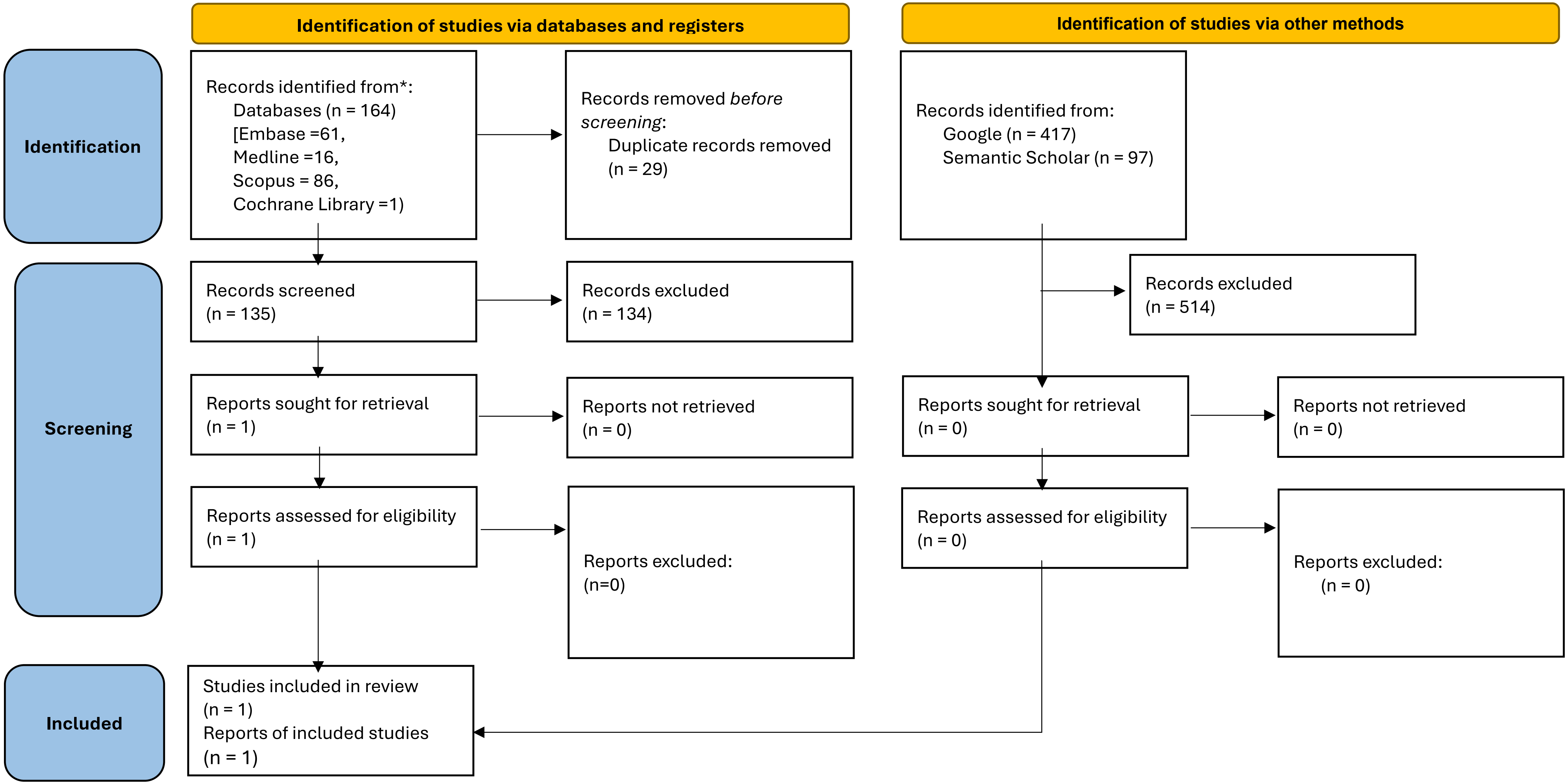

Figure 2. The PRISMA flow chart of the cost-effectiveness of vitrectomy surgery for primary iERMs
