# Supplementary material for: The Visual Effectiveness and Cost‐Effectiveness of Vitrectomy and Membrane Peeling for Primary Idiopathic Epiretinal Membranes (iERMs): A Systematic Review
Source: J Ophthalmol. 2026 Jan 4;2026:5546933. doi: 10.1155/joph/5546933 (PMC12767011; doi:10.1155/joph/5546933)
Supplement: Supplementary file 3 — Supporting Information 3 Appendix file 3: Median BCVA after surgery. [file JOPH-2026-5546933-s002.pdf]

## Appendix 3 Median BCVA after vitrectomy surgery for iERMs

**Table 1 Median BCVA after vitrectomy surgery for iERMs**

| Study/Year | Median BCVA | |
|---|---|---|
| | **Baseline** | **Follow-up point*** |
| **3 months** | | |
| Mieno et al. (2020) | 0.2 (range: 0.1 to 0.4) | 0.1 (range: 0 to 0.02) |
| **6 months** | | |
| Shahzadi et al. (2016) | 0.4 (IQR: 0.22) | 0.185 (IQR: 0.30) |
| Mieno et al. (2020) | 0.2 (range: 0.1 to 0.4) | 0.1 (0 to 0.2) |
| Khanna et al. (2022) | 0.4 (range: 0.1 to 1.2) | 0.1 (range: -0.1 to 0.8) |
| **12 months** | | |
| Mieno et al. (2020) | 0.2 (range: 0.1 to 0.4) | 0.1 (0 to 0.2) |
| **24 months** | | |
| Khanna et al. (2022) | 0.4 (range: 0.1 to 1.2) | 0.1 (range: -0.1 to 0.7) |

* The median BCVA improved significantly compared with median BCVA at baseline
