# Supplementary material for: The Visual Effectiveness and Cost‐Effectiveness of Vitrectomy and Membrane Peeling for Primary Idiopathic Epiretinal Membranes (iERMs): A Systematic Review
Source: J Ophthalmol. 2026 Jan 4;2026:5546933. doi: 10.1155/joph/5546933 (PMC12767011; doi:10.1155/joph/5546933)
Supplement: Supplementary file 4 — Supporting Information 4 Appendix file 4: Data extraction of included studies for the effectiveness of vitrectomy surgery for iERM. [file JOPH-2026-5546933-s009.pdf]

Appendix 4: Data extraction for included studies for the effectiveness of vitrectomy surgery for iERMs

Table 1: Data extraction of included studies of the effectiveness of ERM surgery (1)

**Studies no.**	1	2	3
**Authors**	Tari et al.	Schweitzer et al.	Okamoto et al.
**Year**	2007	2009	2009
**Funder**	The National Eye Institute (EY02115 and EY09076), an unrestricted grant from Research to Prevent Blindness, Inc. (New York, NY), and a grant from The Starr Foundation.	Not reported	Not reported
**Methods**			
**Study design/settings**	Prospective, observational case series, hospital	Prospective, observational case series, hospital	Prospective, observational case series, hospital
**Eyes or unit of randomisation/unit of analysis**	One eye included in study	One eye included in study	One eye included in study
**Participants**			
**Country**	United States	France	Japan
**Total number of participants**<br>**Number (%) of men**	10 patients with iERM<br>6 (60%) men	26 of 57 patients were iERM<br>17 (65%) men	28 patients with iERM<br>13 (46%) men
**Average age ± SD**	63.9 ± 9.5	71.7 ± 8.7	66.7 ± 8.5
**Inclusion criteria**	Patients with iERM	Patients with iERM	Patients with defined as iERM
**Exclusion criteria**	Participants with the presence of significant media opacities and/or other ocular diseases	Not reported	Patients with previous history of vitrectomy surgery and opthalmic disorders except for mild refractive errors and mild cataract. Eyes with secondary ERM
**Interventions**			
**Intervention**	Three-port pars plana vitrectomy and membrane peeling (data on gauge is not reported)	Three-port 23-gauge	Three-port 20-gauge

**Comparator**	No comparator	No comparator	Aged-match normal controls (n=26) for VRQoL only
**Outcomes**			
**Primary outcomes (lists)**	Visual acuity (VA) (logMAR)	Visual acuity (measured by the Monoyer scale) and it was converted into the logarithm of logMAR	Visual acuity (BCVA: logMAR) and vision-related quality of life (VRQoL)
**Secondary outcomes (lists)**	Standard automated achromatic perimetry, OCT, fundus photography and multifocal electroretinography (mfERG)	Anatomical clinical exam performed by OCT and the astigmatism	Letter contrast sensitivity, metamorphopsia, central macular thickness (CMT)
**Adverse events reported (Y/N)**	No	Yes	Yes
**Length follow-up and intervals at which outcomes assessed**	3 months postoperative, all outcomes were measured	1 month postoperative, all outcomes were measured	3 months postoperative, all outcomes were measured
**Included public and patient involvement?**	Not reported	Not reported	Not reported
**Used Patient reported outcome measures (PROMs)?**	No	No	Yes, NEI VFQ-25
**Key findings**	All participants had improvement in VA after surgery. The mean VA significantly changed from 0.40 ± 0.11 to 0.19 ± 0.13 logMAR (p≤0.002).	The mean VA of participants with iERM was improved from 0.53 ± 0.27 to 0.33 ± 0.25 logMAR at 1 month postoperatively.	• The mean of logMAR BCVA was improved significantly from 0.495 ± 0.293 to 0.245 ± 0.294 at 3 months post operatively for intervention group (p<0.001).<br>• The pre-operative VRQoL was significantly lower in patients with iERM compared with normal controls (p<0.001).<br>• iERM surgery significantly improved NEI VFQ-25 composite score (p<0.001). The postoperative NEI VFQ-25 composite score demonstrated significant correlation with the

			severity of metamorphopsia and logMAR BCVA at post operation.
**Note**	9 out of 10 eyes both iERM and internal limiting membrane (ILM) were peeled and identified. One eye of iERM was peeled without ILM identified.	All of 26 patients with iERM were successfully peeled iERM without any complications during and after surgery.	• 57.1% of patients gained 2 or more ETDRS lines. 39.3% gained no change in logMAR BCVA. 3.6% of patient reported vision decrease.<br>• There were no significant intraoperative and postoperative complications. 96.4% patients had ERM surgery on their worse-seeing eye.<br>• There were significantly improvement of letter contrast sensitivity, metamorphopsia and CMT between pre- and post-operatively.

Table 1: Data extraction of included studies of the effectiveness of ERM surgery (2)

Studies no.	4	5	6
**Authors**	Kinoshita et al.	Matsuoka et al.	Kim et al.
**Year**	2012	2012	2013
**Funder**	Not reported	Not reported	None
**Methods**			
**Study design/settings**	Prospective, consecutive case series, hospital	Prospective, case series, hospital	Prospective, single-centred case series, hospital
**Eyes or unit of randomisation/unit of analysis**	One eye included in study	One eye included in study	One eye included in study
**Participants**			
**Country**	Japan	Japan	South Korea
**Total number of participants Number (%) of men**	49 patients with iERM<br>21 (43%) men	26 patients with iERM<br>10 (38%) men	52 eyes of 52 patients with iERM<br>24 (46%) men
**Average age ± SD**	70.2 ± 1.2	70 ± 9	62.1 ± 7.5
**Inclusion criteria**	Patients with iERM	Patients with iERM	Patients with iERM
**Exclusion criteria**	Secondary ERM, absence of preoperative metamorphopsia, other macular diseases, BCVA of worse than 1.0 logMAR unit at baseline, previous intraocular surgery except for uncomplicated cataract surgery, moderate or severe cataract that affected visual acuity	Secondary ERM, eyes with a history of previous intraocular surgeries, eyes with uveitis and vitreoretinal diseases	ocular inflammation, diabetic retinopathy, hypertensive retinopathy and retinal vasculitis, see full details of exclusion criteria in the full paper
**Interventions**			
**Intervention**	25-gauge	25-gauge	23-gague
**Comparator**	No	No	No
**Outcomes**			
**Primary outcomes (lists)**	BCVA (logMAR)	BCVA (logMAR) and VRQoL	BCVA (ETDRS)
**Secondary outcomes (lists)**	Metamorphopsia, OCT	CMT and metamorphopsia	Metamorphopsia and thickness of retinal layers

**Adverse events reported (Y/N)**	No	Yes	No
**Length follow-up and intervals at which outcomes assessed**	1,3,6,9 and 12 months post operation. All outcomes were measured.	3 and 12 months after surgery. All outcomes were measured.	2 and 6 months after surgery. All outcomes were measured.
**Included public and patient involvement?**	Not reported	Not reported	Not reported
**Used Patient reported outcome measures (PROMs)?**	No	Yes, NEI VFQ-25	No
**Key findings**	• The mean preoperative BCVA (logMAR) was 0.38 ± 0.03 (SE). After surgery, the mean BCVA was improved significantly to 0.20 ± 0.03 at 1 month after surgery (p<0.001).<br>• The mean BCVA was significantly improved to 0.09 ± 0.03 at 12 months postoperative (p<0.001).<br>• The BCVA improved by ≥0.2 logMAR units in 67.3% patients and remained unchanged in 32.7% patients.	• The mean BCVA (logMAR) ± SE at baseline, 3 and 12 months postoperatively were 0.41 ± 0.05, 0.17± 0.04, and 0.10± 0.03, respectively. There is significantly different between mean BCVA at baseline and 12 months follow up (P < 0.0001).<br>• The mean BCVA improved > 0.1 logMAR unit in 73% of patients at 3 months and 88% at 12 months. The VFQ-25 composite scores were improved significantly at 3 and 12 months after surgery.<br>• There are difference significantly between baseline and 12 months follow-up for general vision, near activities, distance activities, mental health, role difficulties, and composite score.	The mean BCVA at preoperative, 2 months and 6 months postoperatively were 68.8 ± 8.5, 73.9 ± 7.8, and 76 .0± 7.2 letters, respectively. The BCVA improved throughout follow up points (p < 0.001).
**Note**	• The mean BCVA, metamorphopsia scores for horizontal lines (MH) and vertical lines (MV) improved significantly at 12 months after operation.<br>• The BCVA at 12 months was significantly correlated with the	• The BCVA, CMT and metamorphopsia scores were improved at 3 months and improved more at 12 months after surgery.<br>• At month 12, the post-operative improvement in the	The M-scores at 6 months postoperative follow-up visits was decreased significantly compared with the preoperative value.

	MH or MV scores at 12 months. The baseline MV scores was significantly correlated with the 12-month BCVA and the improvement in the BCVA.	metamorphopsia score was correlated with the improvements in general vision, near activities, distance activities, and the composite scores.<br>• 85% of patients were underwent cataract surgery combined with vitrectomy.<br>• There were no significant intraoperative and postoperative complications	

Table 1: Data extraction of included studies of the effectiveness of ERM surgery (3)

**Studies no.**	7	8	9
**Authors**	Kofod et al.	Shahzadi et al.	Takabatake et al.
**Year**	2016	2016	2018
**Funder**	The Synoptik Foundation. The Danish Agency for Science, Technology and Innovation: FSS09-065546. The Bagenkop Nielsen Eye Foundation	Not reported	None
**Methods**			
**Study design/settings**	RCT	Prospective observational study (case series)	Prospective observational study
**Eyes or unit of randomisation/unit of analysis**	One eye included in study	One eye included in study	One eye included in study
**Participants**			
**Country**	Denmark	Pakistan	Japan
**Total number of participants**<br><br>**Number (%) of men**	53 eyes of 53 patients with symptomatic iERM<br>Surgery: 35% men<br>Watchful waiting: 48% men	30 eyes of 30 patients with iERM stage 2<br>13 men (43.3%)	45 eyes of 45 patients with unilateral iERM<br>16 males (36%)
**Average age ± SD**	Surgery: 69 ± 3<br>Watchful waiting: 66 ± 3	72 ± 5	64.8 ± 8.6
**Inclusion criteria**	Patients with symptomatic iERM in one eye with duration less than 24 months	Patients with iERM stage 2	Patients with unilateral iERM
**Exclusion criteria**	Other significant retinal conditions such as age-related maculopathy worse than hard drusen, any diabetic retinopathy or prior intraocular surgery apart from cataract surgery	Other types of epiretinal membrane (like traumatic ERM, ERM associated with retinal tears, pseudohole type ERM); previous vitreoretinal surgery; corneal opacities; additional ocular comorbidity like glaucoma and concomitant or previous retinal vascular diseases	ERM in both eyes, secondary ERM due to retinal vascular disease and uveitis, optic nerve disease such as glaucoma, visually significant cataracts, a history of intraocular surgery other than uncomplicated cataract surgery, anisometropia greater than 2.0 diopters, and BCVA <0.2 (20/100) because metamorphopsia cannot be

			measured correctly with poor BCVA. Patients with unreliable responses in either the M-CHARTS or NAT measurement or with low-quality OCT images.
**Interventions**			
**Intervention**	23-gauge	23-gauge	Small-gauge pars plana vitrectomy (data on gauge is not reported)
**Comparator**	Watchful waiting	No	No
**Outcomes**			
**Primary outcomes (lists)**	BCVA using ETDRS protocol with the chart at a distance of 4m	The improvement of BCVA (logMAR) of at least two or more lines on ETDRS chart as compared to pre-operative BCVA	BCVA (logMAR) with a 5-meter Landolt chart
**Secondary outcomes (lists)**	Central macular thickness and the number of patients that crossed over from watchful waiting arm to the surgery arm due to deteriorated symptoms	Anatomical outcomes: foveal thickness on SD-OCT	Metamorphopsia using M-CHARTS, and aniseikonia using the New Aniseikonia Test and spectral domain optical coherence tomography (OCT) parameters (macular retina layer thickness and microstructure of the outer retina)
**Adverse events reported (Y/N)**	Yes	Yes	No
**Length follow-up and intervals at which outcomes assessed**	Surgery: 1, 3-, 6-, 9- and 12-months post-operation;<br>Watchful waiting: 3,6,9,12-month post-operation	3 and 6 months after surgery<br>BCVA was only observed at preoperative and 6 months after surgery	6- and 12-months post-surgery
**Included public and patient involvement?**	Not reported	Not reported	Not reported
**Used Patient reported outcome measures (PROMs)?**	No	No	No

**Key findings**	Mean BCVA after 12 months in the watchful waiting group (81 letters) were not significantly different from patients undergoing immediate surgery (82.5 letters p=0.647). The gain was driven by the eight eyes that crossed over to surgery group.	The median (IQR) preoperative BCVA was logMAR 0.4 (0.22), whereas postoperative BCVA was logMAR 0.185 (0.30) with significant (p-value =0.001).	The mean logMAR BCVA at baseline, 6- and 12-months post-surgery were 0.17 ± 0.05 (20/30), -0.01 ± 0.04 (20/20), -0.01 ± 0.04 (20/20), respectively. A significant difference was found compared with baseline (P<0.001).
**Note**	• Both the immediate surgery group and watchful waiting group gained in visual acuity during the study, and the gain was statistically significant.<br>• During 1 year-follow up, 8 patients in watchful waiting (24%) crossed over to surgery and these patients gained a mean of 3.1 letters (SEM 1.38).<br>• Visual acuity gain after surgery is slow and gradual until 9 months postoperatively.<br>• There were no significant complications due to vitrectomy, such as retinal detachment or infectious endophthalmitis.<br>• One patient developed a chronic minimal cystoid macular oedema unresponsive to anti-inflammatory eye drops, but BCVA was unaffected.	• At the end of follow-up period, BCVA was improved in 23 patients (76.7%), stable in 5 patients (16.7%) and decreased in 2 patients (6.7%).<br>• No preoperative and intraoperative complications were found.<br>• In the follow-up period, 70% eyes presented cataract progression at an average time of 3 months after surgery.<br>• The most frequent complication was recurrence of ERM (13.3%). However, these recurrences did not affect BCVA and none of these participants underwent repeat surgery.<br>• Regarding postoperative complications, retinal detachment occurred in one (3.3%) eye after one month of surgery because of an iatrogenic retinal tear formation.<br>• No severe complications such as postoperative endophthalmitis, inflammation, hypotony or	BCVA and horizontal metamorphopsia improved significantly from 6 months after surgery, whereas aniseikonia decreased significantly only at 12 months. Vertical metamorphopsia remained unchanged.

		vitreous haemorrhage were observed.	

Table 1: Data extraction of included studies of the effectiveness of ERM surgery (4)

**Studies no.**	10	11	12
**Authors**	Mieno et al.	Khanna et al.	Hollaus et al.
**Year**	2020	2022	2023
**Funder**	None	Not reported	Not reported
**Methods**			
**Study design/settings**	Prospective observational study	Prospective observational study	Prospective observational study
**Eyes or unit of randomisation/unit of analysis**	Both eyes included in study	One eye included in study	One eye included in study
**Participants**			
**Country**	Japan	France	Austria
**Total number of participants**<br><br>**Number (%) of men**	42 eyes of 40 patients with iERM and symptomatic metamorphopsia (2 with bilateral ERM, 38 with unilateral ERM)<br>17 male (42%)	42 eyes of 42 patients with unilateral iERM<br><br>24 men (58%)	48 eyes of 48 patients with iERM<br><br>24 male (50%)
**Average age ± SD**	69 ± 2.5	72.7 ± 7.4	70 ± 6.7
**Inclusion criteria**	Patients with iERM and symptomatic metamorphopsia	Patients with unilateral iERM	Patients with iERM
**Exclusion criteria**	Patients with a history of vitreoretinal surgery, secondary to ERM, amblyopia or other ophthalmic disorders affecting visual acuity, such as glaucoma, severe cataracts, keratoconus, retinal vascular occlusion and other macular disorders	Patients with ERM secondary to an inflammatory or vascular ocular pathology or appearing as a result of retinal detachment or retinal tear, co-existing pathology (except cataracts) of the eye affected by ERM or the fellow eye, history of strabismus or amblyopia, and absence of normal retinal correspondence; history of vitreoretinal surgery, bilateral ERM, distance BCVA ⩽ 4/10 in the fellow eye, refractive anisometropia defined by a difference in spherical equivalent >2 diopsters between both eyes; axial anisometropia	Any additional diseases causing a decrease in vision apart from cataract, i.e. glaucoma, age-related macular degeneration, any vascular disease and diabetic retinopathy. Patients with opacities of the optical media causing a decrease in OCT images quality as well as not receiving endotamponade during surgery were not included

Interventions			
**Intervention**	25- or 27-gauge	25-gauge	23-gauge
**Comparator**	No	No	No
**Outcomes**			
**Primary outcomes (lists)**	BCVA (logMAR) measured with a standard Japanese Landolt VA chart	Distance monocular and binocular BCVA measured with the Monoyer decimal scale then converted to LogMAR, and vision-related quality of life with the use of NEI VFQ-25	BCVA converted to logMAR with Snellen equivalent
**Secondary outcomes (lists)**	Reading ability (MNREAD-J) and metamorphopsia score	Horizontal and vertical metamorphopsia, horizontal and vertical aniseikonia, stereoacuity, central retinal thickness and NEI VFQ-25	Mean photoreceptor thickness (PRT), central retinal thickness (CRT)
**Adverse events reported (Y/N)**	No	No	No
**Length follow-up and intervals at which outcomes assessed**	3,6-, and 12-months post-operation	6 months and 2 years post-operation	1 week, 1 month and 3 months after surgery
**Included public and patient involvement?**	Not reported	Not reported	Not reported
**Used Patient reported outcome measures (PROMs)?**	No	Yes, NEI VFQ-25	No
**Key findings**	• The median logMAR BCVA at baseline, 3,6,12 months postoperative were 0.2 (0.1–0.4), 0.1 (0–0.2), 0.1 (0–0.2), 0.1 (0–0.2).<br>• When compared to baseline values, 19 eyes showed an improvement of 0.2 logMAR or more in BCVA at 12 months post-surgery, while 23 eyes showed a change of 0.1 logMAR or less in BCVA at 12 months post-surgery. No eye exhibited a worsening of	• The monocular distance BCVA in the operated eye (LogMAR), median (range) at pre-op, post-op 6 months, and post-op 12 months were 0.4 (0.1 to 1.2), 0.1 (− 0.1 to 0.8), and 0.1 (− 0.1 to 0.7).	BCVA was 0.30 ± 0.24 logMAR (Snellen equivalent approximately 20/40) at baseline and improved to 0.25 ± 0.17 logMAR (Snellen equivalent approximately 20/36; p = 0.151) at week 1, 0.24 ± 0.17 logMAR (Snellen equivalent approximately 20/35; p = 0.070) at month 1 and 0.15 ± 0.16 logMAR (Snellen equivalent approximately 20/28; p < 0.001) at month 3.

	0.2 logMAR or more in BCVA at 12 months post-surgery.<br>• Subgroup analysis of the 23 eye with a change of 0.1 logMAR or less in BCVA at 12 months post-surgery revealed no change in median BCVA.		
**Note**	• Significant improvement in median BCVA, RA and CPS (critical print size) were observed at 3 months post-surgery and these improvements were retained at each subsequent post-surgery follow-up examination.<br>• In all patients, the ERM was anatomically removed, and no intra- or post-surgery complications were observed.	• At 6 months post-operatively, distance monocular BCVA, horizontal metamorphopsia and the composite score of NEI-VFQ-25 significantly improved, in comparison to baseline.<br>• At 2 years post-operatively, distance monocular and binocular BCVA, horizontal and vertical metamorphopsia and the composite score of NEI-VFQ-25 significantly improved, in comparison to baseline.<br>• Removal of uiERM improves VR QoL and achieve good visual outcomes on both monocular and binocular visual parameters over long-term.<br>• No patients presented a recurrence of ERM during follow-up on SD-OCT.	• BCVA increased significantly from baseline to 3-months follow-up (0.3 logMAR - 0.15 logMAR, Snellen equivalent approximately 20/40-20/28 respectively).<br>• There was no correlation between baseline PRT and BCVA at any visit after surgery, nor between PRT and BCVA at any visits.
