# Supplementary material for: The Visual Effectiveness and Cost‐Effectiveness of Vitrectomy and Membrane Peeling for Primary Idiopathic Epiretinal Membranes (iERMs): A Systematic Review
Source: J Ophthalmol. 2026 Jan 4;2026:5546933. doi: 10.1155/joph/5546933 (PMC12767011; doi:10.1155/joph/5546933)
Supplement: Supplementary file 5 — Supporting Information 5 Appendix file 5: Quality and risk of bias assessment of included studies for the effectiveness of vitrectomy surgery for iERM. [file JOPH-2026-5546933-s004.pdf]

Appendix 5. Quality and risk of bias assessment for included studies for the effectiveness of vitrectomy surgery for iERMs

Table 1: JBI Critical Appraisal Checklist for Case Series studies (1)

**Checklists/study**	**Tari et al. (2007)**	**Okamoto et al. (2008)**	**Schweitzer et al. (2009)**	**Kinoshital et al. (2012)**	**Matsuoka et al. (2012)**	**Kim et al. (2012)**	**Shahzadi et al. (2016)**
Were there clear criteria for inclusion in the case series?	Yes	Yes	Yes	Yes	Yes	Yes	Yes
Was the condition measured in a standard, reliable way for all participants included in the case series?	Unclear	Yes	Unclear	Unclear	Yes	Unclear	Yes
Were valid methods used for identification of the condition for all participants included in the case series?	Unclear	Yes	Unclear	Unclear	Yes	Unclear	Yes
Did the case series have consecutive inclusion of participants?	No	Yes	Yes	Yes	Yes	Yes	Unclear
Did the case series have complete inclusion of participants?	No	Yes	Yes	Yes	Yes	Yes	Yes
Was there clear reporting of the demographics of the participants in the study?	Yes	Yes	Yes	Yes	Yes	Yes	Yes
Was there clear reporting of clinical information of the participants?	Yes	Yes	Yes	Yes	Yes	Yes	Yes
Were the outcomes or follow up results of cases clearly reported?	Yes	Yes	Yes	Yes	Yes	Yes	Yes
Was there clear reporting of the presenting site(s)/clinic(s) demographic information?	Yes	Yes	Yes	Yes	Yes	Yes	Yes
Was statistical analysis appropriate?	Yes	Yes	Yes	Yes	Yes	Yes	Yes
**Overall appraisal (include/exclude/seek further info)**	**Include**	**Include**	**Include**	**Include**	**Include**	**Include**	**Include**

Table 1: JBI Critical Appraisal Checklist for Case Series studies (2)

**Checklists/study**	**Takabatake et al. (2018)**	**Mieno et al. (2020)**	**Khanna et al. (2022)**	**Hollaus et al. (2023)**
Were there clear criteria for inclusion in the case series?	Yes	Yes	Yes	Yes
Was the condition measured in a standard, reliable way for all participants included in the case series?	Unclear	Yes	Yes	Unclear
Were valid methods used for identification of the condition for all participants included in the case series?	Yes	Yes	Yes	Unclear
Did the case series have consecutive inclusion of participants?	Yes	Yes	Unclear	Unclear
Did the case series have complete inclusion of participants?	Yes	Yes	Yes	Yes
Was there clear reporting of the demographics of the participants in the study?	Yes	Yes	Yes	Yes
Was there clear reporting of clinical information of the participants?	Yes	Yes	Yes	Yes
Were the outcomes or follow up results of cases clearly reported?	Yes	Yes	Yes	Yes
Was there clear reporting of the presenting site(s)/clinic(s) demographic information?	Yes	Yes	Yes	Yes
Was statistical analysis appropriate?	Yes	Yes	Yes	Yes
**Overall appraisal (include/exclude/seek further info)**	**Include**	**Include**	**Include**	**Include**

Table 2: JBI Critical Appraisal Tool for RCTs

**Assessor:** HK and KD	**Date of Appraisal:** 02/10/2024	**Record Number:**
**Study Author:** Kofod et al.	**Study Title:** Deferral of surgery for epiretinal membranes: is it safe? Results of a randomised controlle trial	**Study Year:** 2015

**Internal Validity**		**Choice - Comments/Justification**	**Yes**	**No**	**Unclear**	**N/A**
**Bias related to selection and allocation**						
1	**Was true randomization used for assignment of participants to treatment groups?**	Random block size and list generated by masked assistant in Microsoft excel	☒	☐	☐	☐
2	**Was allocation to treatment groups concealed?**	Masked assistant	☒	☐	☐	☐
3	**Were treatment groups similar at the baseline?**		☒	☐	☐	☐
**Bias related to administration of intervention/exposure**						
4	**Were participants blind to treatment assignment?**		☐	☒	☐	☐
5	**Were those delivering the treatment blind to treatment assignment?**		☐	☒	☐	☐
6	**Were treatment groups treated identically other than the intervention of interest?**	Patients in the watchful waiting group could cross over to the surgery arm if they actively requested surgery or if their visual acuity had reduced from baseline by ≤ 10 ETDRS letters.	☐	☒	☐	☐
**Bias related to assessment, detection and measurement of the outcome**						
7	**Were outcome assessors blind to treatment assignment?**		**Yes**	**No**	**Unclear**	**N/A**
	**Outcome 1** BCVA measurement	measurements were performed by trained optometrists masked to randomisation	☒	☐	☐	☐
8	**Were outcomes measured in the same way for treatment groups?**		**Yes**	**No**	**Unclear**	**N/A**
	**Outcome 1** BCVA measurement	visual acuity was measured using ETDRS protocol with the chart at a distance of 4m for both groups	☒	☐	☐	☐

			Yes	No	Unclear	N/A
9	**Were outcomes measured in a reliable way**					
	**Outcome 1** BCVA measurement	visual acuity was measured using ETDRS protocol with the chart at a distance of 4m + measurement performed by trained optometrists	☒	☐	☐	☐

Bias related to participant retention

10	**Was follow up complete and if not, were differences between groups in terms of their follow up adequately described and analysed?**					
	**Outcome 1**		**Yes**	**No**	**Unclear**	**N/A**
	Result 1: gain in visual acuity	Two patients did not complete the 12-month follow up, the results of last observations from the 9-month visit was carried forward in data analysis.	☒	☐	☐	☐
	Result 2		☐	☐	☐	☐
	Result 3		☐	☐	☐	☐
	Result 3		☐	☐	☐	☐

Statistical Conclusion Validity

11	**Were participants analysed in the groups to which they were randomized?**					
	**Outcome 1**		**Yes**	**No**	**Unclear**	**N/A**
	Result 1: gain in visual acuity	Eight patients in watchful waiting crossed over to surgery group at 12 months – gains were driven by the eyes that crossed over to surgery	☐	☒	☐	☐
	Result 2		☐	☐	☐	☐
	Result 3		☐	☐	☐	☐

12	**Was appropriate statistical analysis used?**					
	**Outcome 1**		**Yes**	**No**	**Unclear**	**N/A**
	Result 1	Parametric and non-parametric approach used Bonferroni adjustment for multiple comparisons	☒	☐	☐	☐
	Result 2		☐	☐	☐	☐

			Yes	No	Unclear	N/A
	Result 3		☐	☐	☐	☐

			Yes	No	Unclear	N/A
13	**Was the trial design appropriate and any deviations from the standard RCT design (individual randomization, parallel groups) accounted for in the conduct and analysis of the trial?**	Parallel groups<br>Deviation: crossover of patients – analysed and adjusted	☒	☐	☐	☐

Overall appraisal: **Include:** ☒ **Exclude:** ☐ **Seek Further Info:** ☐

Comments:

Table 3. Risk of bias assessment for RCT crossover trial study

The development of the RoB 2 tool was supported by the MRC Network of Hubs for Trials Methodology Research (MR/L004933/2- N61), with the support of the host MRC ConDuCT-II Hub (Collaboration and innovation for Difficult and Complex randomised controlled Trials In Invasive procedures - MR/K025643/1), by MRC research grant MR/M025209/1, and by a grant from The Cochrane Collaboration.

This work is licensed under a Creative Commons Attribution-NonCommercial-NoDerivatives 4.0 International License.

Study details

**Reference**	Kofod et al. (2015)

Study design

- ☐ Individually-randomized parallel-group trial
- ☐ Cluster-randomized parallel-group trial
- X Individually randomized cross-over (or other matched) trial

For the purposes of this assessment, the interventions being compared are defined as

Experimental: Immediate surgery Comparator: Watchful waiting

**Specify which outcome is being assessed for risk of bias**	Mean BCVA after 12 months
**Specify the numerical result being assessed.** In case of multiple alternative analyses being presented, specify the numeric result (e.g. RR = 1.52 (95% CI 0.83 to 2.77) and/or a reference (e.g. to a table, figure or paragraph) that uniquely defines the result being assessed.	Mean BCVA after 12 months in the watchful waiting group (81 letters) were not significantly different from patients undergoing immediate surgery (82.5 letters, p=0.647).

Is the review team's aim for this result...?

☑ to assess the effect of *assignment to intervention* (the 'intention-to-treat' effect)
☐ to assess the effect of *adhering to intervention* (the 'per-protocol' effect)

If the aim is to assess the effect of *adhering to intervention*, select the deviations from intended intervention that should be addressed (at least one must be checked):
☐ occurrence of non-protocol interventions
☐ failures in implementing the intervention that could have affected the outcome
☐ non-adherence to their assigned intervention by trial participants

Which of the following sources were obtained to help inform the risk-of-bias assessment? (tick as many as apply)
☑ Journal article(s) with results of the trial
☐ Trial protocol
☐ Statistical analysis plan (SAP)
☐ Non-commercial trial registry record (e.g. ClinicalTrials.gov record)
☐ Company-owned trial registry record (e.g. GSK Clinical Study Register record)
☐ "Grey literature" (e.g. unpublished thesis)
☐ Conference abstract(s) about the trial
☐ Regulatory document (e.g. Clinical Study Report, Drug Approval Package)
☐ Research ethics application
☐ Grant database summary (e.g. NIH RePORTER or Research Councils UK Gateway to Research)
☐ Personal communication with trialist
☐ Personal communication with the sponsor

Risk of bias assessment

Responses underlined in green are potential markers for low risk of bias, and responses in red are potential markers for a risk of bias. Where questions relate only to sign posts to other questions, no formatting is used.

Domain 1a: Risk of bias arising from the randomization process

Signalling questions	Comments	Response options
**1.1 Was the allocation sequence random?**	Randomisation was conducted using a random block size and list generated by masked assistant in Microsoft Excel.	Y / PY / PN / N / NI
**1.2 Was the allocation sequence concealed until participants were enrolled and assigned to interventions?**		Y / PY / PN / N / NI
**1.3 Did baseline differences between intervention groups at the start of the first period suggest a problem with the randomization process?**	The median age of participants in immediate surgery group was higher than watchful waiting by 2.5 years (p value = 0.045). The other baseline characteristics were well balance between groups (p >0.1).	Y / PY / PN / N / NI
**Risk-of-bias judgement**		Low / High / Some concerns
Optional: What is the predicted direction of bias arising from the randomization process?		NA / Favours experimental / Favours comparator / Towards null /Away from null / Unpredictable

Domain S: Risk of bias arising from period and carryover effects

Signalling questions	Comments	Response options
**S.1 Was the number of participants allocated to each of the two sequences equal or nearly equal?**	Patients were randomised 1:2 to immediate surgery or to watchful waiting.	Y/PY/PN/N/NI
**S.2 If N/PN/NI to S.1: Were period effects accounted for in the analysis?**		NA/Y/PY/PN/N/NI
**S.3 Was there sufficient time for any carryover effects to have disappeared before outcome assessment in the second period?**	There was no carry effects because there was no intervention in watchful waiting group.	Y/PY/PN/N/NI
**Risk-of-bias judgement**		Low / High / Some concerns
Optional: What is the predicted direction of bias arising from period and carryover effects?		NA / Favours experimental / Favours comparator / Towards null /Away from null / Unpredictable

Domain 2: Risk of bias due to deviations from the intended interventions (effect of assignment to intervention)

Signalling questions	Comments	Response options
**2.1. Were participants aware of their assigned intervention during each period of the trial?**		Y / PY / PN / N / NI
**2.2. Were carers and people delivering the interventions aware of participants' assigned intervention during each period of the trial?**		Y / PY / PN / N / NI
**2.3. If Y/PY/NI to 2.1 or 2.2: Were there deviations from the intended intervention that arose because of the trial context?**		NA / Y / PY / PN / N / NI
**2.4 If Y/PY to 2.3: Were these deviations likely to have affected the outcome?**		NA / Y / PY / PN / N / NI
**2.5. If Y/PY/NI to 2.4: Were these deviations from intended intervention balanced between groups?**		NA / Y / PY / PN / N / NI
**2.6 Was an appropriate analysis used to estimate the effect of assignment to intervention?**	Intention-to-treat	Y / PY / PN / N / NI
**2.7 If N/PN/NI to 2.6: Was there potential for a substantial impact (on the result) of the failure to analyse participants in the group to which they were randomized?**		NA / Y / PY / PN / N / NI
**Risk-of-bias judgement**		Low / High / Some concerns
Optional: What is the predicted direction of bias due to deviations from intended interventions?		NA / Favours experimental / Favours comparator / Towards null /Away from null / Unpredictable

Domain 2: Risk of bias due to deviations from the intended interventions (effect of adhering to intervention)

Signalling questions	Comments	Response options
**2.1. Were participants aware of their assigned intervention during each period of the trial?**		Y / PY / PN / N / NI
**2.2. Were carers and people delivering the interventions aware of participants' assigned intervention during each period of the trial?**		Y / PY / PN / N / NI
**2.3. [If applicable:] If Y/PY/NI to 2.1 or 2.2: Were important non-protocol interventions balanced between interventions?**		NA / Y / PY / PN / N / NI
**2.4. [If applicable:] Were there failures in implementing the intervention that could have affected the outcome?**		NA / Y / PY / PN / N / NI
**2.5. [If applicable:] Was there non-adherence to the assigned intervention regimen that could have affected participants' outcomes?**		NA / Y / PY / PN / N / NI
**2.6. If N/PN/NI to 2.3, or Y/PY/NI to 2.4 or 2.5: Was an appropriate analysis used to estimate the effect of adhering to the intervention?**		NA / Y / PY / PN / N / NI
**Risk-of-bias judgement**		Low / High / Some concerns
Optional: What is the predicted direction of bias due to deviations from intended interventions?		NA / Favours experimental / Favours comparator /

		Towards null /Away from null / Unpredictable

Domain 3: Risk of bias due to missing outcome data

Signalling questions	Comments	Response options
**3.1 Were data for this outcome available for all, or nearly all, participants randomized?**	Two patients did not complete the 12-month examination and in these two cases last observation from the 9-month visit was carried forward in the data analysis.	Y / PY / PN / N / NI
**3.2 If N/PN/NI to 3.1: Is there evidence that the result was not biased by missing outcome data?**		NA / Y / PY / PN / N
**3.3 If N/PN to 3.2 Could missingness in the outcome depend on its true value?**		NA / Y / PY / PN / N / NI
**3.4 If Y/PY/NI to 3.3: Is it likely that missingness in the outcome depended on its true value?**		NA / Y / PY / PN / N / NI
**Risk-of-bias judgement**		Low / High / Some concerns
Optional: What is the predicted direction of bias due to missing outcome data?		NA / Favours experimental / Favours comparator / Towards null /Away from null / Unpredictable

Domain 4: Risk of bias in measurement of the outcome

Signalling questions	Comments	Response options
**4.1 Was the method of measuring the outcome inappropriate?**		Y / PY / PN / N / NI
**4.2 Could measurement or ascertainment of the outcome have differed between interventions within each sequence?**	Participants in immediate surgery groups were follow up one more visit (after one-month post surgery) than waiting watchful group. However, the main outcome (mean BCVA) was 12-months after surgery.	Y / PY / PN / N / NI
**4.3 If N/PN/NI to 4.1 and 4.2: Were outcome assessors aware of the intervention received by study participants?**		NA / Y / PY / PN / N / NI
**4.4 If Y/PY/NI to 4.3: Could assessment of the outcome have been influenced by knowledge of intervention received?**		NA / Y / PY / PN / N / NI
**4.5 If Y/PY/NI to 4.4: Is it likely that assessment of the outcome was influenced by knowledge of intervention received?**		NA / Y / PY / PN / N / NI
**Risk-of-bias judgement**		Low / High / Some concerns
Optional: What is the predicted direction of bias in measurement of the outcome?		NA / Favours experimental / Favours comparator / Towards null /Away from null / Unpredictable

Domain 5: Risk of bias in selection of the reported result

Signalling questions	Comments	Response options
**5.1 Were the data that produced this result analysed in accordance with a pre-specified analysis plan that was finalized before unblinded outcome data were available for analysis?**		Y / PY / PN / N / NI
**Is the numerical result being assessed likely to have been selected, on the basis of the results, from...**		
**5.2. ... multiple eligible outcome measurements (e.g. scales, definitions, time points) within the outcome domain?**	There is only one possible way in which the outcome domain can be measured (hence there is no opportunity to select from multiple measures	Y / PY / PN / N / NI
**5.3 ... multiple eligible analyses of the data?**		Y / PY / PN / N / NI
**5.4 Is a result based on data from both periods sought, but unavailable on the basis of carryover having been identified?**	Answer 'N' if data from both periods contribute to the result being assessed for risk of bias.	Y / PY / PN / N / NI
**Risk-of-bias judgement**		Low / High / Some concerns
Optional: What is the predicted direction of bias due to selection of the reported result?		NA / Favours experimental / Favours comparator / Towards null /Away from null / Unpredictable

Overall risk of bias

**Risk-of-bias judgement**		Low / High / Some concerns
Optional: What is the overall predicted direction of bias for this outcome?		NA / Favours experimental / Favours comparator / Towards null /Away from null / Unpredictable

This work is licensed under a Creative Commons Attribution-NonCommercial-NoDerivatives 4.0 International License.
