# Supplementary material for: The Visual Effectiveness and Cost‐Effectiveness of Vitrectomy and Membrane Peeling for Primary Idiopathic Epiretinal Membranes (iERMs): A Systematic Review
Source: J Ophthalmol. 2026 Jan 4;2026:5546933. doi: 10.1155/joph/5546933 (PMC12767011; doi:10.1155/joph/5546933)
Supplement: Supplementary file 6 — Supporting Information 6 Appendix file 6: Quality assessment for the included study for the cost‐effectiveness of vitrectomy surgery for iERM. [file JOPH-2026-5546933-s005.pdf]

Appendix 6. Quality assessment for included study for the cost-effectiveness of vitrectomy surgery for iERMs

Table 1: JBI Critical Appraisal Checklist for economic evaluation

No.	Checklists	Gupta et al. (2008)
1	Is there a well-defined questions?	Yes
2	Is there comprehensive description of alternatives?	Yes
3	Are all important and relevant costs and outcomes for each alternatives identified?	Yes
4	Has clinical effectiveness been established?	Yes
5	Are costs and outcomes measured accurately?	No
6	Are costs and outcomes valued credibly?	No
7	Are costs and outcomes adjusted for differential timing?	Yes
8	Is there an incremental analysis of costs and consequences?	Yes
9	Were sensitivity analyses conducted to investigate uncertainty in estimates of cost or consequences?	Yes
10	Do study results include all issues of concern to users?	Yes
11	Are the results generalizable to setting of interest in the review	No

Table 2: CHEERS 2022 Checklist

	Item	Guidance for Reporting	Reported insection
**TITLE**			
Title	1	Identify the study as an economic evaluation and specify theinterventions being compared.	Title, p.923
**ABSTRACT**			
Abstract	2	Provide a structured summary that highlights context, key methods,results and alternative analyses.	Abstract, p.923
**INTRODUCTION**			
Background andobjectives	3	Give the context for the study, the study question and its practicalrelevance for decision making in policy or practice.	Introduction, p.923
**METHODS**			
Health economic analysis plan	4	Indicate whether a health economic analysis plan was developed and where available.	Not reported
Study population	5	Describe characteristics of the study population (such as age range, demographics, socioeconomic, or clinical characteristics).	Methods, p.924
Setting and location	6	Provide relevant contextual information that may influence findings.	Methods, p.924-925
Comparators	7	Describe the interventions or strategies being compared and why chosen.	Methods, p.924-925
Perspective	8	State the perspective(s) adopted by the study and why chosen.	Methods, p.924
Time horizon	9	State the time horizon for the study and why appropriate.	Methods, p.924
Discount rate	10	Report the discount rate(s) and reason chosen.	Methods, p.925
Selection of outcomes	11	Describe what outcomes were used as the measure(s) of benefit(s)and harm(s).	Methods, p.924
Measurement ofoutcomes	12	Describe how outcomes used to capture benefit(s) and harm(s)were measured.	Methods, p.924-925
Valuation of outcomes	13	Describe the population and methods used to measure and value outcomes.	Methods, p.924-925
Measurement and valuation of resources and costs	14	Describe how costs were valued.	Methods, p.924
Currency, price date,and conversion	15	Report the dates of the estimated resource quantities and unit costs, plus thecurrency and year of conversion.	Methods, p.924
Rationale and description of model	16	If modelling is used, describe in detail and why used. Report if the model is publicly available and where it can be accessed.	Methods, p.924-925
Analytics and assumptions	17	Describe any methods for analysing or statistically transforming data, anyextrapolation methods, and approaches for validating any model used.	Methods, p.924-925
Characterizing heterogeneity	18	Describe any methods used for estimating how the results of the studyvary for sub-groups.	Methods, p.924-925
Characterizing distributional effects	19	Describe how impacts are distributed across different individuals or adjustments made to reflect priority populations.	Results, p.925-926
Characterizing uncertainty	20	Describe methods to characterize any sources of uncertainty in the analysis.	Methods, p.924-925

Approach to engagement with patients and others affected by the study	21	Describe any approaches to engage patients or service recipients, the general public, communities, or stakeholders (e.g., clinicians or payers) inthe design of the study.	Not reported
**RESULTS**			
Study parameters	22	Report all analytic inputs (e.g., values, ranges, references) includinguncertainty or distributional assumptions.	Results, p.925-926
Summary of mainresults	23	Report the mean values for the main categories of costs and outcomes ofinterest and summarise them in the most appropriate overall measure.	Results, p.925-926
Effect of uncertainty	24	Describe how uncertainty about analytic judgments, inputs, or projections affect findings. Report the effect of choice of discount rate and time horizon,if applicable.	Results, p.925-926
Effect of engagement with patients and othersaffected by the study	25	Report on any difference patient/service recipient, general public, community,or stakeholder involvement made to the approach or findings of the study	Not reported
**DISCUSSION**			
Study findings, limitations, generalizability, andcurrent knowledge	26	Report key findings, limitations, ethical or equity considerations not captured,and how these could impact patients, policy, or practice.	Discussion, p.926-927
Source of funding	27	Describe how the study was funded and any role of the funder inthe identification, design, conduct, and reporting of the analysis	Acknowledgement, p.927
Conflicts of interest	28	Report authors conflicts of interest according to journal or International Committee of Medical Journal Editors requirements.	Conflicts of interest, p.927
